# New-Onset Diabetes Mellitus Presenting As Diabetic Ketoacidosis in Patients With COVID-19: A Case Series

**DOI:** 10.7759/cureus.16290

**Published:** 2021-07-10

**Authors:** Aysha Sarwani, Mahmood Al Saeed, Husain Taha, Rawdha M Al Fardan

**Affiliations:** 1 Endocrinology, Salmaniya Medical Complex, Manama, BHR; 2 Internal Medicine, Salmaniya Medical Complex, Manama, BHR

**Keywords:** covid-19, diabetic ketoacidosis, sars‐cov‐2, type 1 diabetes mellitus (t1d), diabetes mellitus type 2

## Abstract

Coronavirus disease 2019 (COVID‐19), a 2020 pandemic, has been linked to another global health problem, the diabetes pandemic. Both are existing in a bi-directional association. COVID-19 has been shown to be associated with worse outcomes in those with pre-existing diabetes mellitus. Nevertheless, recent data have emerged highlighting the inter-relationship between new-onset diabetes mellitus and COVID-19. Here, we present four cases admitted to the hospital with newly diagnosed diabetes mellitus associated with COVID-19. We aim to review the available literature regarding the complex association between COVID-19 and new-onset diabetes, causative factors and triggers, treatment strategies, outcomes, and its burden on the health system in general.

## Introduction

It has been one year since coronavirus disease 2019 (COVID-19) has been identified and it continues to affect all aspects of life including health, economy, education, lifestyle, and most notably leading to unexpected loss of lives. COVID-19 presents with its diverse range of clinical presentations, ranging from a non-progressive symptom-free course to a more aggressive course leading to death [1]. At the same time, the extent of organ involvement can be unpredictable. COVID-19 exhibits increased morbidity and mortality as the person advances with age, especially in those with comorbidities, such as diabetes mellitus (DM) [2]. Currently, the data on the long-term influence of COVID-19 on endocrinological disease are limited [3]. The current literature, in terms of long-term effects, is more informative about previous coronavirus outbreaks, which were associated with new occurrences of DM; for instance, the severe acute respiratory syndrome coronavirus (SARS-CoV) [3]. It has been proven that coronavirus infections have a substantial effect on DM management due to the inflammation and altered immune system responses caused by it, leading to difficulties in glycemic control [4].

Globally, depending on the region, 20%-50% of patients diagnosed with COVID-19 also suffer from DM [5]. Unfortunately, there are limited data on the correlation of newly diagnosed DM in COVID-19 patients. Here, we describe four patients who presented to our hospital with Diabetic Ketoacidosis (DKA) as the initial manifestation of both, newly diagnosed DM and COVID-19. It is plausible that COVID-19 unveiled underlying DM in these patients by exacerbating the condition or related to pancreatic organ involvement due to the infection. This will allow us to examine the link between DM and COVID-19 and the impact of this co-occurrence on the health system as a whole.

## Case presentation

Case 1

A 23‐year‐old man was referred to our hospital due to high blood glucose readings. He initially presented to a private clinic with fatigue, polydipsia, body aches, and weight loss for two weeks. He denied any history of fever, respiratory, or gastrointestinal symptoms. Family and past medical histories were unremarkable. He had no previous medical record for any hemoglobin A1c (HbA1c) or blood glucose readings. His temperature was 37.0 °C, with a respiratory rate of 20 breaths per minute, heart rate of 81 beats per minute, blood pressure of 118/78 mmHg, and oxygen saturation of 99% on room air. He had no respiratory distress. The lungs were bilaterally clear to auscultation with normal cardiovascular and abdominal exams. His body mass index (BMI) was 18.3 kg/m^2^.

**Figure 1:**
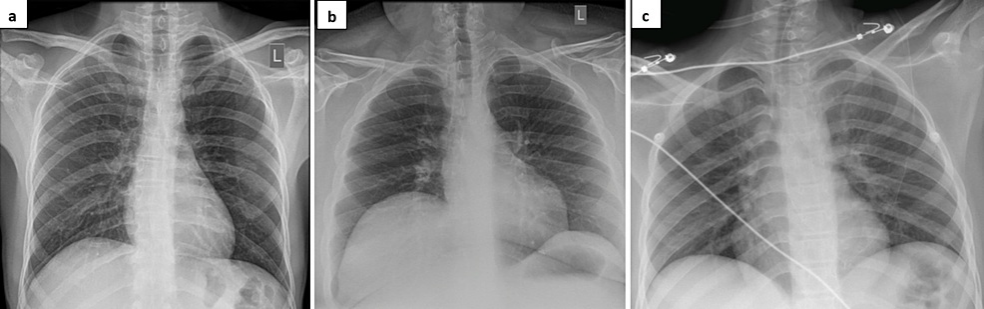
Chest X-ray images demonstrating the absence of features of COVID-19 pneumonia for case 1 (a), case 3 (b), and case 4 (c).

Serological investigations revealed hyperglycemia, high anion gap metabolic acidosis, and ketonemia (Table 1). Urinalysis was positive for glucose and ketones; screening for severe acute respiratory syndrome coronavirus 2 (SARS‐CoV‐2) reverse‐transcription polymerase chain reaction (RT‐PCR) returned positive results. Insulin antibodies, islet cell antibodies, anti‐cyclic citrullinated peptide, and antibody glutamic acid decarboxylase were not conducted. He was diagnosed and treated as DKA as per hospital protocol with intravenous fluids and continuous insulin infusion at 0.1 unit/kg/hour with hypokalemia correction. No specific COVID‐19 treatment was given as his chest x-ray was normal (Figure 1). The DKA resolved on the following day and he was started on a subcutaneous insulin regimen. On the fifth day of admission, the patient noted an abscess in the sole of his right foot and gave a history of stepping on a nail 10 days prior to admission. He developed a localized collection with pain and tenderness over it. There was no bony involvement and he underwent incision and drainage of the abscess. The HbA1c sample requested on admission came to be 14.8%, indicating pre-existing DM.

**Table 1:** Blood results for the cases described in the report. HbA1C - hemoglobin A1c

Test	Case 1	Case 2	Case 3	Case 4	Normal values	Unit
Random blood sugar	41	24	23	37.6	3.6-8.9	mmol/L
pH	7.19	7.15	6.9	6.9	7.32-7.42	-
Bicarbonate	15.7	10.3	5.2	7	22-29	mmol/L
Urea	5.7	3.4	4.1	14.8	3.2-8.2	mmol/L
Creatinine	59.00	29	57	108	44-97	μmol/L
Sodium	130	132	133	150	132-146	mmol/L
Potassium	4.5	5.1	4.3	3.7	3.5-5.5	mmol/L
Chloride	96	103	104	118	98-107	mmol/L
Serum Ketones	+	+	+	+	-	-
White blood cells	5.8	6.28	14.8	21.65	3.6-9.6	x10^9/L
Neutrophils	58.7	57	84	86.3	42.2-75.2	%
Lymphocytes	34.90	35	13	9	20.5-55.1	%
Anion gap	23	21	28	28	8-12	mEq/L
HbA1C	14.8	7.4	N/A	12.2	<6.5	%
Procalcitonin	0.04	0.1	0.42	0.95	0-0.5	μg/L

His repeated COVID-19 RT-PCR tests on days 5 and 9 were both negative. He was discharged home on day 9 with a subcutaneous insulin basal-bolus regimen (insulin glargine 30 units once daily and insulin aspart 10 units three times daily). There were no respiratory complications throughout hospitalization.

Case 2

A 27‐year‐old woman presented to our hospital with a history of fever after being in close contact with a COVID-19 patient. She had a history of gestational diabetes in the previous year. Her family history was significant for type 2 DM from the first degree. There were initially no respiratory tract or gastrointestinal symptoms. Her temperature was 38.5 °C, with a respiratory rate of 18 breaths per minute, heart rate of 87 beats per minute, blood pressure of 125/82 mmHg, and oxygen saturation of 97% on room air. There was no respiratory distress and the lungs were bilaterally clear to auscultation. The cardiac and abdominal exams were also normal. Her BMI was 21.7 kg/m^2^. COVID-19 RT‐PCR was positive. She was admitted to the hospital due to the lack of proper home self-isolation.

**Figure 2:**
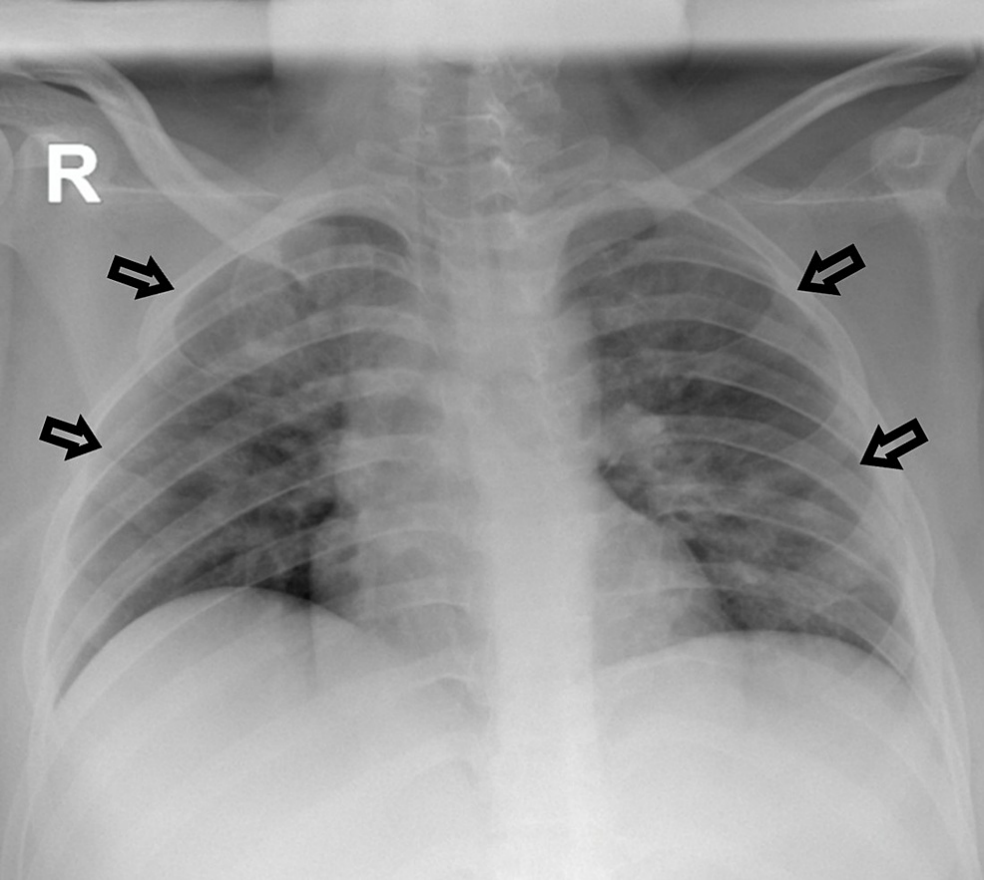
Chest x-ray of case 2 showing bilateral infiltrates consistent with COVID-19 pneumonia.

On the second day of admission, she developed episodes of vomiting associated with dry cough and dyspnea. She was still pyrexial at 38 °C, oxygen saturations were maintained at 97% on room air, and the respiratory rate was 25 breaths per minute. Her chest was clear on auscultation and cardiovascular and abdominal exams were unremarkable. Serological investigations revealed a high anion gap metabolic acidosis, hyperglycemia with a random blood glucose of 24 mmol/L, and ketonemia (Table 1). She denied any weight loss, polyuria, or polydipsia. She was diagnosed with DKA and treated as per hospital protocol with intravenous fluids and continuous insulin infusion at 0.1 unit/kg/hour with hypokalemia correction. She was also initiated on antibiotic therapy with ceftriaxone and azithromycin as her chest x-ray revealed bilateral infiltrates (Figure 2). After resolution of the DKA, she was shifted to a subcutaneous insulin regimen on day 3. On day 10, she completed the antibiotic course and was discharged home on subcutaneous insulin (insulin glargine 20 units once daily and insulin aspart 10 units three times daily) with a negative RT-PCR swab result.

Case 3

A 27‐year‐old man came to our hospital complaining of a week’s history of polydipsia and polyuria associated with nausea and vomiting without respiratory or other gastrointestinal symptoms. His medication history was insignificant except for using an energy-boosting supplement to improve his physical performance at the gym. His past medical history was only significant for glucose-6-phosphate dehydrogenase (G6PD) reduced activity. His BMI was 39 kg/m^2^. Vitally, he was tachycardic with a pulse of 124 beats per minute, hypertensive with a blood pressure of 163/86 mmHg, and his oxygen saturation was 98% on room air. His temperature was 37.0 °C. Cardiovascular, respiratory, and abdominal examinations were normal. Blood test results revealed hyperglycemia, ketonemia, and high anion gap metabolic acidosis (Table 1). Urinalysis showed glucosuria and ketonuria. His routine SARS‐CoV‐2 RT-PCR came back positive and his chest x-ray was normal (Figure 1). He was diagnosed with DKA and treated as per hospital protocol with intravenous fluids and continuous insulin infusion at 0.1 unit/kg/hour with hypokalemia correction. During admission, he noted an abscess in the groin region and after review by the surgical team he was started on amoxicillin and clavulanic acid. He remained in DKA for four days and was then shifted to a subcutaneous insulin regimen. His blood pressure subsequently normalized and did not develop any complications related to COVID-19. His repeated RT-PCR came back negative on day 7 and he was discharged home on subcutaneous insulin glargine 36 units once daily, insulin aspart 14 units three times daily, and oral metformin 500mg three times daily with follow up for the dressing of his abscess.

Case 4

A 14-year-old boy presented to our hospital complaining of abdominal pain, nausea and vomiting for three days followed by fever for one day. He denied any respiratory or other gastrointestinal symptoms. There was no significant past medical history. His temperature was 37.8 °C, with a respiratory rate of 21 breaths per minute, heart rate of 85 beats per minute, blood pressure of 108/58 mmHg, and oxygen saturation of 99% on room air. He had no respiratory distress and the lungs were bilaterally clear to auscultation. The cardiac and abdominal exams were not significant apart from mild generalized abdominal tenderness. His BMI was 20.1 kg/m^2^. His family history was unremarkable. Serological investigations demonstrated hyperglycemia, high anion gap metabolic acidosis, and ketones in the serum and urine (Table 1). His routine SARS‐CoV‐2 RT-PCR came back positive. He was diagnosed with new-onset DM presenting with DKA and COVID-19. His chest x-ray was normal (Figure 1). The patient was reviewed by the surgical team due to abdominal pain and underwent computed tomography of the abdomen which did not reveal any abnormalities. Treatment was initiated as per hospital protocol with intravenous fluids and continuous insulin infusion at 0.1 units/kg/hour with hypokalemia correction. The DKA resolved after two days and the patient was started on a basal-bolus subcutaneous insulin regimen (insulin glargine 20 units once daily and insulin aspart 15 units three times daily). He tested positive for islet cell antigen 2 (ANTI-IA2) antibodies with readings of 112.3 IU/mL (<10) and insulin autoantibodies (IAA) reading of 1 ++ U/mL (<0.40). His repeat SARS‐CoV‐2 RT-PCR at day 10 came back negative.

## Discussion

Poor outcomes and increased mortality rates have been demonstrated when COVID-19 coexists with DM [6]. Until now, the relationship between DM and COVID-19 is unclear. Papadokostaki et al. highlighted several important issues regarding the linked relationship between COVID-19 and DM [3]. They briefly theorized the possibility of COVID-19 leading to new-onset DM secondary to beta-cell infection by SARS-CoV-2. This may not only lead to new-onset DM but may unveil already existing DM due to disturbances in the cycle of glucose metabolism [7]. Li et al. also pointed out that all patients with newly diagnosed DM are more likely to have elevated levels of inflammatory markers as well as indicators of multi-organ injury, which in turn, may lead to a serious course of COVID-19 [7].

We came across four cases of new-onset DM presenting with DKA who were admitted with COVID-19 at our institute. Similar cases have been reported in the literature since the start of the pandemic. A case of DKA as the presentation of DM and COVID-19 was reported by Chee et al. in a 37-year-old patient [8]. This patient seemed to have previously undiagnosed DM exacerbated by COVID-19 infection leading to DKA as evidenced by his high HbA1c (Table 2). This is similar to three of our cases where the HbA1c was elevated indicating a similar course of events.

**Table 2:** Summary of case studies reporting new-onset diabetes mellitus in COVID-19 cases.

Reference	Case	Age (years), sex	Symptoms	PMH	Diagnosis	HbA1c (<6.5%)	Complications	Length of Stay (Days)	Outcome
Chee et al. [8]	1	37, Male	Fever, vomiting, polydipsia, polyuria	None	Diabetic ketoacidosis as new-onset Diabetes Mellitus	-	none	-	Discharged on subcutaneous insulin.
Heany et al. [9]	1	54, Male	Dyspnea, fatigue, cough	Hypertension, Renal calculi, Testicular hypofunction	Diabetic ketoacidosis as new-onset Diabetes Mellitus	-	none	5	Discharged on subcutaneous insulin.
Suwanwongse & Shabarek [10]	1	18, Male	Fatigue, polydipsia, polyuria	none	Diabetic ketoacidosis as new-onset Diabetes Mellitus	10.4	none	3	Discharged on Metformin and subcutaneous insulin.
	2	51, Male	Fatigue, anorexia, polydipsia, polyuria	none	Diabetic ketoacidosis as new-onset Diabetes Mellitus	12.4	none	3	Discharged on subcutaneous insulin.
	3	64, Female	Polydipsia, polyuria	Breast cancer	Hyperglycemia as new-onset Diabetes Mellitus	-	none	-	Discharged on metformin.
Otair et al. [11]	1	47, Male	Fatigue, polyuria	None	Diabetic ketoacidosis as new-onset Diabetes Mellitus	6.2	Pneumonia	16	Discharged on gliclazide, linagliptin, and metformin.
Rabizadeh et al. [12]	1	16, Male	Fatigue, dyspnea, nausea, polyuria, polydipsia, abdominal pain.	None	Diabetic ketoacidosis as new-onset Diabetes Mellitus	12.9	None	-	Discharged on subcutaneous insulin.
Benyakhlef et al. [13]	1	3, Male	Dyspnea, Fatigue, vomiting, Polyuria, Polydipsia	None	Diabetic ketoacidosis as new-onset Diabetes Mellitus	10.3	Pneumonia	10	Discharged on subcutaneous insulin.

In another case reported by Heaney et al., a 54-year-old patient who presented with shortness of breath was found to have COVID-19 with DKA and was thus newly diagnosed with DM [9]. The presence of multiple comorbidities including hypertension and obesity might be associated with undiagnosed diabetes; however, the HbA1c was not available. He progressed well without any complications. In a case series reported by Suwanwongse and Shabarek, three cases of newly diagnosed DM were associated with COVID-19, of which, two patients were diagnosed with DKA and the other came in with hyperglycemia after being diagnosed with COVID-19 [10]. Rabizadeh et al. and Benyakhlef et al. both reported cases of young patients with new-onset DM presenting as DKA; the latter being complicated by pneumonia [12,13].

The causal co-existence between COVID-19 and DM is still unclear. As seen by multiple case reports, patients who are newly diagnosed with DM are prone to developing diabetic emergencies of DKA and hyperosmolar hyperglycemic state (HHS) requiring insulin therapy [11,12]. A mechanism by which SARS-CoV-2 leads to abnormalities of glucose metabolism and destruction of pancreatic beta cells has been suggested [14]. It is proposed that by binding to angiotensin-converting enzyme 2 (ACE-2) receptors, which are numerous in pancreatic beta and adipose tissue cells at the level of cell entry, the SARS-CoV-2 leads to ketosis-prone diabetes. This could be the causative factor of DM in COVID-19 patients [15]. Apart from this, the atypical immune reaction caused by the SARS-CoV-2 may precipitate an autoimmune destructive process targeting the pancreatic beta islet cells and leading to the development of DM [15]. Two of our patients (cases 1 and 3) presented with abscesses in different areas. To that effect, given the absence of COVID-19-related symptoms, a bacterial infection could be the precipitating factor. In a review by Syangtan et al., it was concluded that about half of SARS-CoV-2 infected patients were asymptomatic at the time of screening and therefore are considered as asymptomatic carries [16]. Those were predominantly female and children. It is, therefore, more likely for those patients (cases 1 and 3) to have been asymptomatic carriers of COVID-19 which was picked up incidentally on pre-admission screening. There is also the possibility of a false positive result which should be kept in mind especially in asymptomatic cases. Multiple factors have been identified as culprits including sampling errors, contamination, cross-reaction with other viruses, and sample mix-ups [17]. It is common practice at our institute to not repeat a positive result until reaching the fifth day from symptom onset which is a major limitation in our case series.

The baseline high HbA1c revealed at the time of diagnosis of DM suggests that the diagnoses of new-onset DM along with COVID-19 may be a result of metabolic disturbances caused by the latter which unveiled pre-existing DM rather than actually causing it. Nonetheless, we are witnessing a high occurrence of DKA as the first presentation of DM with the concurrent diagnosis of COVID-19; therefore, detailed studies to investigate the association between DM and COVID-19 are required.

All cases of new-onset DM in COVID-19 mentioned in the literature so far were associated with good outcomes. On the contrary, it is known that pre-existing DM and COVID-19 have a negative bidirectional relationship with unfavorable outcomes [15,18]. This could be related to the short duration of the newly diagnosed disease in case reports; moreover, the poor outcomes of patients previously diagnosed with DM in larger studies could be due to co-morbid conditions and preexisting disease complications. To that effect, physicians need to be acquainted with the relationship between COVID-19 and DM highlighting the importance of screening COVID-19 patients for DM regardless of the past medical history [19]. Early diagnosis will lead to optimal treatment in addition to less complications and lower mortality [20].

Comorbidities, such a DM, lead to poorer clinical outcomes with a higher chance of admission to the intensive care unit alongside higher mortality rates. [21,22]. This could be due to the fact that people with DM have impaired innate and adaptive immune responses leading to a state of chronic and low-grade inflammation that can suddenly result in an abrupt alteration of systemic metabolism [22]. In addition to that, Bode et al. studied 1122 COVID-19 inpatients and concluded that patients with diabetes and/or uncontrolled hyperglycemia spent a long time in hospital (5.7 vs 4.3 days, P < 0.001) [23]. These poorer outcomes will, in theory, lead to an absolute increase in costs and burden on the healthcare system.

## Conclusions

COVID-19 is a public health emergency with many unknowns in terms of its treatment and outcomes. Our case series describe four cases of DKA as the presenting symptom of newly diagnosed DM with COVID-19. All of the patients had favorable outcomes in terms of recovery from COVID-19 and all required subcutaneous insulin regimens upon discharge. The HbA1c levels on admission suggest the presence of undiagnosed DM and that DKA was the result of an immune modulated effect of the SARS-CoV-2 infection. It is important that emergency physicians screen patients for DM including fasting blood glucose and HbA1c once the diagnosis of COVID-19 is confirmed regardless of the presenting complaint. At the same time, any symptom suggesting DKA and underlying diabetes should be promptly investigated. More research is needed for this interrelationship, pathogenesis, disease pattern, and prognosis.
